# Regional variation and factors associated with average expenditure per headache disorder hospitalization in Brazil: a macrocosting analysis in the SUS (2008–2023)

**DOI:** 10.11606/s1518-8787.2026060007119

**Published:** 2026-06-15

**Authors:** Jesuély Spieckert de Souza, Vanise Grassi, Marcos Otávio Brum Antunes, Mariana Cristina Ribeiro, Vinicius da Silva Lessa de Oliveira, Adriel Brandão, João José Freitas de Carvalho, Frederico Friedrich

**Affiliations:** I Pontifícia Universidade Católica do Rio Grande do Sul. Escola de Medicina. Programa de Pós-Graduação em Pediatria e Saúde da Criança. Porto Alegre, RS, Brazil; II Santa Casa de Porto Alegre. Serviço de Neurologia. Porto Alegre, RS, Brazil; III Centro Universitário Christus. Faculdade de Medicina. Fortaleza, CE, Brazil

**Keywords:** Headache, Migraine, Hospitalization Expenditures, Epidemiology, Brazil, Health Systems

## Abstract

**OBJECTIVE:**

To quantify the total expenditure on hospitalizations due to headache disorders within the Brazilian Unified Health System (SUS) and to identify the sociodemographic, clinical, and regional factors associated with the average expenditure per hospitalization.

**METHODS:**

This observational study used SIH-SUS data from 2008 to 2023. Expenditures were inflation-adjusted and categorized by sex, age, and ICD-10 diagnosis. Given the extreme positive asymmetry of the expenditure data, a Generalized Linear Model with a Gamma family and log link function was used to identify adjusted expenditure multipliers.

**RESULTS:**

Total expenditure reached BRL 77,643,809.55. The highest volume was concentrated among females (65.7%) and the 19–59 age group (66.1%). The Generalized Linear Model revealed that patients aged 60 years or older had a 21% higher expenditure multiplier (multiplier = 1.21) per event. The South Region showed a structurally 73.7% higher expenditure multiplier (Multiplier = 1.737) per event, even after adjustment for complexity. Vascular headache not elsewhere classified (247.2% higher) and status migrainosus (124.8% higher) were the costliest diagnoses. Crude rate analysis showed the South Region with the highest utilization rate (108.0 hospitalizations per 100,000 inhabitants).

**CONCLUSION:**

The high adjusted expenditure multipliers in the South/Southeast and the long median stays in the North/Northeast point to structural inequalities in the cost and timeliness of care provision. Public policies must reinforce specialized outpatient management to reduce high-cost hospitalizations and promote equitable resource allocation.

## INTRODUCTION

Migraine is the most disabling neurological disorders among children and adolescents and ranks second among adults, surpassed only by stroke. Moreover, it is the second most prevalent disorder in terms of years lived with disability^1^. Headaches, whether primary or secondary, comprise multiple subtypes, including tension-type headache (TTH), migraine, and cluster headache^2^. Migraine has a global prevalence of approximately 15%, with a higher prevalence among women. TTH affects 42% of the global population, and both disorders have considerable socioeconomic repercussions^3^. Cluster headache is estimated to have a global prevalence of 0.12%, and, like other types of headaches, it is associated with significant impairments in the affected individuals^4^.

In Brazil, it is estimated that 70.6% of the population experiences the symptom of headache over the past 12 months, with 15.8% associated with migraine and 29.5% with TTH^5^. Individuals who suffer from migraine endure debilitating symptoms such as pulsating pain, photophobia, phonophobia, nausea, vomiting, and pain aggravated by routine physical activities. These symptoms often lead to the disruption of daily activities, which directly impacts on the productivity of the economically active population affected by these disorders. During migraine attacks, approximately 19% of individuals need to take time off work, 45% feel apprehensive about driving due to the diagnosis, 90% of spouses assume additional household tasks, and 94% of their children’s routine activities are affected.

Hospitalizations registered by the Brazilian Unified Health System (SUS) due to migraine and other headache syndromes totaled 42.9 thousand from 2014 to 2018^7^. A supplementary study, considering all aspects of care, analyzed a period of four years and identified a total of 50.3 thousand hospitalizations, of which 94.32% (47.5 thousand) were emergency admissions^8^.

The high prevalence of the disease reflects financial impacts. In Europe, it is estimated that annual expenses for the treatment of migraine, including direct and indirect costs, amount to 27 billion euros. In North America, the expenses reach an average annual of 4.1 thousand dollars per individual in the United States for the treatment of chronic migraine and 1.9 thousand dollars in Canada^10^. In Brazil, in 2003, the total cost of expenses generated by the public health system for the treatment of migraine was estimated at 140.4 million dollars, at which time the exchange rate was R$ 1.51 per US$ 1^11^.

Given the impacts generated by hospitalizations and the Brazilian economy, understanding the dynamics of headache hospitalizations in Brazil and their associated expenditures has become a public health issue. Accordingly, the present study utilized an extensive dataset covering the period from 2008 to 2023 from the Hospital Information System (SIH/SUS) to address this gap. The main objective was to quantify the total expenditure on hospitalizations due to headaches within the SUS and to identify the sociodemographic, clinical, and regional factors associated with the average expenditure per hospitalization.

## METHODS

### Study Design

This is an observational study with a macrocosting analysis based on secondary data from national, open-access databases. The study is reported in accordance with the items outlined in The Strengthening the Reporting of Observational Studies in Epidemiology (STROBE) Statement^12^.

### Data Sources

Data on hospitalizations and associated expenditures were extracted from the SIH-SUS, available through the Department of Informatics of the Unified Health System (DATASUS)^13^. DATASUS is a public governmental platform that stores and disseminates information related to the Brazilian public health system. SIH-SUS offers nationwide coverage and includes all hospitalizations within the public healthcare system. It is important to emphasize that the expenditure analysis in this study reflects the perspective of the SUS as the payer (federal spending) and therefore does not encompass other costs associated with the economic burden of the disease (e.g., patient out-of-pocket expenses, private sector costs, or indirect productivity losses).

### Population and Sample

The study included all hospitalizations registered in SIH-SUS with primary diagnosis codes for headache disorders according to the International Classification of Diseases, 10th Revision (ICD-10). There were no exclusion criteria, as records with other ICD codes were filtered during preprocessing. Therefore, the population consisted of all hospitalized patients with the selected ICD codes across Brazil from January 2008 to December 2023.

### Data Extraction

Data were extracted using the R software (version 4.3.1) with the Microdatasus package^a^ *,* an open-source tool designed to facilitate data extraction, cleaning, and preprocessing from DATASUS. Inclusion criteria were based on ICD-10. We selected codes G43 and G44, including the following subcategories: G43.0 (Migraine without aura), G43.1 (Migraine with aura), G43.2 (Status migrainosus), G43.3 (Complicated migraine), G43.8 (Other migraine), G43.9 (Migraine, unspecified), G44.0 (Cluster headache syndrome), G44.1 (Vascular headache, not elsewhere classified), G44.2 (Tension-type headache), G44.3 (Chronic post-traumatic headache), G44.4 (Drug-induced headache, not elsewhere classified), and G44.8 (Other specified headache syndromes).

Expenditure data were obtained from the “Total value”, which combines hospital service expenditures and professional service expenditures, considering only SUS reimbursements to hospitals. Values were extracted in Brazilian reais (BRL), without transformation, and were later adjusted for inflation. There were no restrictions regarding age (0–100 years), sex, race/ethnicity, or region. For age-stratified analyses, data were categorized post-collection into: < 18 years, 19–59 years, and ≥ 60 years. Data were extracted across all five Brazilian regions using state codes defined by the *Instituto Brasileiro de Geografia e Estatística* (IBGE - Brazilian Institute of Geography and Statistics)^14^.

Population data used for calculating rates per 100,000 inhabitants were also sourced from the IBGE^14^. Procedure codes related to hospitalizations were retrieved from the *Sistema de Gerenciamento da Tabela de Procedimentos, Medicamentos* e OPM do SUS (SIGTAP – Management System for the SUS Table of Procedures, Medications, and Medical Devices) database. Data extraction included data from January 2008 to December 2023, which represents the most complete and consistently available period on the DATASUS platform. Data extraction was performed in March 2025, and all records were independently reviewed by two researchers to enhance data reliability and quality.

### Expenditure Adjustment for Inflation

To ensure robust expenditure analyses over time, monetary values were adjusted for inflation using December 2023 as the reference month. The adjustment was performed in *R* using the *DeflateBR* package, which corrects monetary values according to Brazil’s official inflation index, the Broad Consumer Price Index (IPCA)^15^. This step enabled a more accurate temporal analysis by controlling for inflationary effects. Additionally, annual total expenditures were normalized by the number of hospitalizations in the same year, and this expenditure-to-admission ratio was stratified by sex and age group. Therefore, part of the expenditures analyses was based on deflated and hospitalization-adjusted values.

### Statistical Analysis

For descriptive analyses, the median and interquartile range (IQR) were used, given the non-normal distribution of the expenditure and length of stay variables. This non-normality was characterized by extreme positive asymmetry (Skewness = 39.822) and high leptokurtosis (Excess Kurtosis = 4,978.808). For median comparisons, the Kruskal–Wallis test was used, followed by Dunn’s *post hoc* test, to compare the median hospital length of stay among ICD codes, regions, and states. This procedure was also used to compare crude expenditure ratios between states. Analyses of sex proportions by ICD code were performed using the chi-square test, applying the Bonferroni correction. To assess temporal trends in average expenditure per hospitalization, a time series approach was adopted. The Durbin-Watson test was first applied to the residuals of a preliminary linear regression model to check for the presence of serial autocorrelation. Upon confirming autocorrelation, the Generalized Least Squares (GLS) Model was used, adjusted with an autoregressive error structure of the first order (AR(1)). This method was essential to obtain valid annual variation coefficients by correcting data serial dependence.

Factors associated with average expenditure per hospitalization were investigated using the Generalized Linear Model (GLM). Given the continuous, highly asymmetric, and positive nature of the expenditure variable, the Gamma family with a log link function was employed. The significance of the regional variables was maintained in the adjusted model to assess the persistence of structural inequalities. Statistical significance was set at p < 0.05.

Since this study is based on open-access databases and does not involve patients or the collection of personal identifiers, ethics committee approval is not required.

## RESULTS

Between 2008 and 2023, the total expenditure on headache-related hospitalizations within the SUS amounted to BRL 77,643,809.55, covering a total of 126,396 hospitalizations. Regarding the expenditure distribution, females accounted for the largest proportion, representing 65.7% (BRL 51,012,455.91) of the total value. By age group, the highest volume of expenditure was concentrated among adults aged 19–59 years (BRL 51,583,669.76; 66.1%), followed by the elderly aged 60 years or more (BRL 16,243,913.57; 20.8%) and children and adolescents under 18 years (BRL 9,816,946.22; 12.6%). However, the analysis of average expenditure per hospitalization showed that the 60 years or older group recorded the highest expenditure per event across all regions (Table 1).

**Table 1:** Descriptive statistics of hospitalizations, mean and median expenditure, and length of stay by region and age group (2008–2023).

Region	Age group	Hospitalizations number	Mean expenditure per hospitalization	Median expenditure (Q1-Q3)	Length of stay, days Median (Q1–Q3)
Central West	18–59	4.840	BRL 350.49	BRL 206.06 (BRL 137.00 – BRL 368.10)	2 (1–4)
Central West	> 60	824	BRL 407.08	BRL 225.97 (BRL 155.67 – BRL 386.95)	2 (2–4)
Central West	< 18	1.053	BRL 321.51	BRL 209.45 (BRL 126.39 – BRL 339.82)	2 (1–3)
Northeast	18–59	21.986	BRL 697.19	BRL 322.01 (BRL 161.58 – BRL 698.03)	3 (2–6)
Northeast	> 60	5.481	BRL 1,023.50	BRL 465.61 (BRL 182.78 – BRL 861.90)	3 (2–7)
Northeast	< 18	4.819	BRL 627.77	BRL 307.02 (BRL 170.24 – BRL 627.64)	2 (2–5)
North	18–59	5.391	BRL 463.35	BRL 204.64 (BRL 147.95 – BRL 466.24)	3 (2–5)
North	> 60	1.256	BRL 1,366.55	BRL 553.10 (BRL 198.60 – BRL 894.17)	5 (3–9)
North	< 18	1.168	BRL 310.70	BRL 187.53 (BRL 147.91 – BRL 264.00)	3 (2–4)
Southeast	18–59	32.605	BRL 540.43	BRL 337.38 (BRL 204.64 – BRL 621.71)	2 (1–4)
Southeast	> 60	5.827	BRL 618.47	BRL 374.84 (BRL 233.38 – BRL 678.95)	2 (1–5)
Southeast	< 18	7.055	BRL 471.17	BRL 298.19 (BRL 188.00 – BRL 510.88)	2 (1–4)
South	18–59	23.620	BRL 655.15	BRL 454.63 (BRL 226.13 – BRL 836.29)	2 (2–4)
South	> 60	6.186	BRL 810.32	BRL 544.45 (BRL 293.80 – BRL 970.40)	2 (2–3)
South	< 18	3.915	BRL 484.26	BRL 326.67 (BRL 199.69 – BRL 609.48)	2 (1–4)

Note: the mean expenditure was calculated as the total deflated expenditure/number of hospitalizations.

### Variation by Diagnosis (ICD-10) and Procedures

The length of hospital stay varied significantly across the country’s regions. The Northeast and North regions recorded the highest median lengths of stay, while the South and Southeast regions showed the shortest durations (Table 1). Among the ICD-10 codes analyzed, G43.2 (Status migrainosus) recorded the highest total expenditure, amounting to BRL 21,811,201.69, followed by G43.3 (Complicated migraine), with BRL 17,131,792.62, and G44.1 (Vascular headache, not elsewhere classified), with BRL 12,996,563.71. These same CIDs recorded the highest medians for expenditure per hospitalization (Table 2).

**Table 2:** Median expenditure and length of stay by ICD-10 diagnosis (2008–2023).

ICD-10	Hospitalizations number	Median expenditure (Q1–Q3)	Length of stay, days Median (Q1–Q3)
G43.0	1,130	BRL 96.17 (BRL 64.19–BRL 204.88)	1 (1–2)
G43.1	599	BRL 151.91 (BRL 63.93–BRL 285.05)	1 (1–2)
G43.2	47,140	BRL 330.65 (BRL 187.53–BRL 637.61)	3 (2–5)
G43.3	49,741	BRL 305.18 (BRL 184.92–BRL 558.22)	2 (2–4)
G43.8	542	BRL 142.03 (BRL 76.45–BRL 280.33)	1.5 (1–3)
G43.9	3,492	BRL 104.94 (BRL 75.47–BRL 227.63)	1 (1–2)
G44	345	BRL 124.09 (BRL 56.04–BRL 251.61)	2 (1–3)
G44.0	482	BRL 603.57 (BRL 289.75–BRL 1,026.75)	3 (1–7)
G44.1	17,072	BRL 787.62 (BRL 563.36–BRL 1,129.18)	3 (2–6)
G44.2	1,653	BRL 144.29 (BRL 71.05–BRL 284.78)	1 (1–2)
G44.3	444	BRL 182.52 (BRL 79.54–BRL 334.78)	1 (1–3)
G44.4	179	BRL 96.24 (BRL 63.12–BRL 245.52)	1 (1–2)
G44.8	3,577	BRL 179.40 (BRL 75.97–BRL 332.51)	1 (1–3)

ICD-10: International Statistical Classification of Diseases–10th Revision; G43.0 (Migraine without aura); G43.1 (Migraine with aura); G43.2 (Status migrainosus); G43.3 (Complicated migraine); G43.8 (Other migraine); G43.9 (Migraine; unspecified); G44 (Other headache syndromes);G44.0 (Cluster headache syndrome); G44.1 (Vascular headache; not elsewhere classified); G44.2 (Tension-type headache); G44.3 (Chronic post-traumatic headache); G44.4 (Drug-induced headache; not elsewhere classified); and G44.8 (Other specified headache syndromes).

Note: values are presented as median (25th–75th percentile).

Regarding the duration of stay, the diagnoses G43.2, G44.0 (Cluster headache), and G44.1 recorded the highest medians (3 days), with wider interquartile ranges, whereas the other diagnoses had a median stay of 1 day. Analysis of procedures showed that “03.03.04.003-3 - Treatment of complicated migraine” was performed in 34,330 hospitalizations related to G43.2 and 41,579 associated with G43.3.

### Association of Sex by the ICD

The chi-square test showed a statistically significant difference in the case distribution for most diagnoses (p < 0.01). The proportion of women (65.7%) was maintained across almost all diagnoses (ranging from 62.66% for G44.1 to 73.06% for G43.8). However, CIDs G44.0 (Cluster headache) and G44.3 (Chronic post-traumatic headache) did not show statistically significant differences between sexes (p > 0.01), with G44.3 recording 51.80% of male cases.

### Temporal Trend Analysis

**Figure 1:**
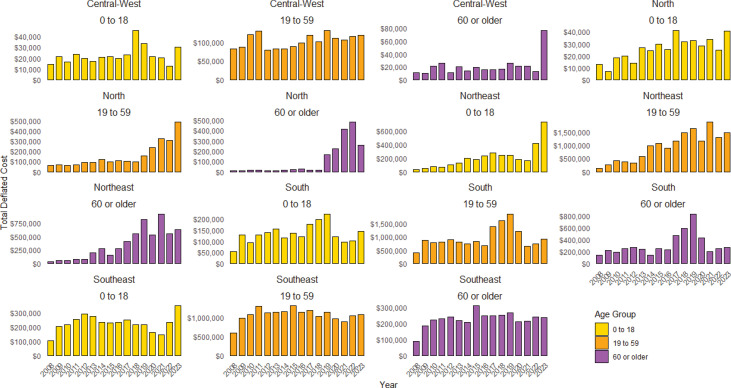
Temporal trend of hospitalization expenditures by age group and region. ICD-10: G43.0 (Migraine without aura); G43.1 (Migraine with aura); G43.2 (Status migrainosus); G43.3 (Complicated migraine); G43.8 (Other migraine); G43.9 (Migraine; unspecified); G44.0 (Cluster headache syndrome); G44.1 (Vascular headache; not elsewhere classified); G44.2 (Tension-type headache); G44.3 (Chronic post-traumatic headache); G44.4 (Drug-induced headache; not elsewhere classified); and G44.8 (Other specified headache syndromes).

The average expenditure per hospitalization over the period (2008–2023) did not present a statistically significant linear trend in the adjusted time series model. The GLS model, corrected for a (AR(1)) after the Durbin-Watson test ({DW} = 1.3113, p = 0.03754), resulted in a non-significant coefficient (beta = -1.1887, p = 0.7349) (Table 3, Figure 1).

**Table 3:** Annual hospitalization expenditures for headache disorders in Brazil (2008–2023) in Brazilian reais (BRL).

	G43.0	G43.1	G43.2	G43.3	G43.8	G43.9	G44.0	G44.1	G44.2	G44.3	G44.4	G44.8
2008	11,598.3	3,355.17	756,689.59	583,489.15	4,997.01	21,864.13	16,596.67	330,543.62	15,317.27	4,051.95	1,413.54	19,665.05
2009	5,603.71	2,824.02	1,408,300.21	1,408,300.21	2,997.88	27,721.63	14,869.78	704,710.51	12,648.33	2,851.60	584.68	28,180.71
2010	7,279.31	2,537.39	1,367,232.62	1,124,838.75	3,979.40	25,122.69	9,977.91	813,115.67	11,699.92	2,594.74	759.39	25,758.45
2011	7,492.98	2,483.45	1,451,553.84	1,254,582.58	3,449.59	30,951.05	14,425.22	947,167.63	21,126.43	3,787.19	896.11	27,328.49
2012	8,283.66	1,020.58	1,449,513.61	1,388,317.30	4,308.05	31,627.84	8,546.03	759,856.59	17,828.25	2,373.83	1,179.77	26,382.50
2013	7,562.41	4,317.84	1,440,381.27	1,299,026.41	4,961.90	28,662.88	15,572.06	1,143,516.01	11,358.20	1,986.48	1,448.59	32,212.77
2014	6,338.73	1,779.86	1,480,640.90	1,540,267.64	4,804.43	34,769.05	16,530.00	1,185,072.87	16,010.67	2,335.34	1,097.11	29,739.31
2015	22,086.63	42,094.67	1,678,004.13	1,773,366.47	10,642.57	68,884.12	13,212.22	1,048,340.80	26,469.01	8,884.54	1,821.15	50,350.26
2016	16,921.85	8,169.31	1,521,240.00	1,647,267.49	17,460.06	51,750.67	25,486.87	956,098.96	23,549.38	13,911.68	1,740.42	55,033.16
2017	26,050.66	37,752.18	1,639,201.48	1,871,831.82	5,994.95	57,675.09	161,998.77	1,861,396.48	42,711.83	14,542.44	4,547.24	141,781.56
2018	20,439.53	27,649.27	2,028,018.98	2,774,732.23	50,145.03	60,125.68	82,481.00	1,247,658.26	30,194.77	17,672.63	5,953.36	140,110.54
2019	17,179.54	19,756.77	3,080,724.07	2,411,686.37	8,573.22	67,803.57	23,459,03	1,937,841.19	39,659.20	7,719.12	5,749.43	153,520.15
2020	45,081.85	15,333.13	2,349,580.10	1,524,380.41	8,174.19	40,185.71	17,744.62	1,394,341.57	25,680.30	19,004.12	2,701.51	136,366.90
2021	7,797.69	31,679.90	3,082,592.27	1,292,503.63	11,483.72	40,095.05	35,511.23	1,307,940.37	31,931.91	17,851.58	6,899.69	180,924.94
2022	22,224.85	19,054.30	2,398,591.94	1,480,575.40	17,583.64	58,069.62	51,259.92	1,463,726.32	35,850.30	29,429.98	46,580.16	185,337.08
2023	15,401.22	18,834.63	3,226,489.52	1,793,119.40	19,502.00	55,836.33	24,709.42	1,328,213.08	35,850.30	28,874.09	89,877.44	174,144.59

Note: data sourced from the SIHSUS (Hospital Information System of the Brazilian Unified Health System), inflation-adjusted. Diagnoses are classified according to the International Classification of Diseases, 10th Revision (ICD-10). All values are reported in Brazilian Reais (BRL).

The analysis of expenditure and hospitalizations adjusted for the resident population (crude rates per 100,000 inhabitants) revealed that the average hospitalization rate for the period was 60.0 hospitalizations per 100,000 inhabitants, and the total expenditure per population was approximately BRL 36,000 per 100,000 inhabitants. The South region recorded the highest crude rates, both in terms of utilization and expenditure, with 108.0 hospitalizations per 100,000 inhabitants and a total expenditure of BRL 71,487 per 100,000 inhabitants. In contrast, the Central-West recorded the lowest rates for both indicators, with BRL 13,750 in expenditure and 39.0 hospitalizations per 100,000 inhabitants. The Northeast presented the second-highest expenditure per population (BRL 42,136/100k inhabitants) and the second-highest volume of hospitalizations (57.0/100 k inhabitants)

**Figure 2:**
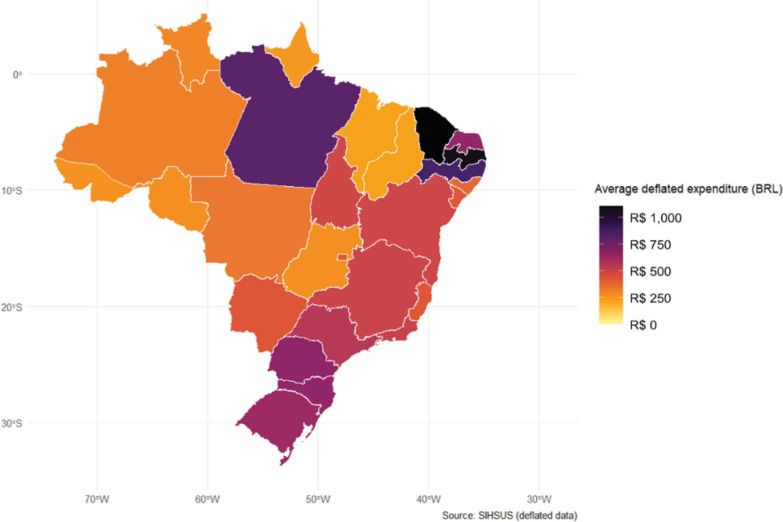
Heatmap of the average cost per hospitalization for diagnoses G43 and G44 and their subclassifications across Brazilian states.

Regarding the state-by-state expenditures in the country (Table 4, Figure 2), São Paulo showed the highest volume of both total expenditure and hospitalizations, with BRL 11,320,760.02 and 30,824 cases, respectively. When analyzing average expenditures, Ceará showed the highest value, registering 9,974 hospitalizations during the period, BRL 8,842,013.26 in total expenditure, and BRL 886.51 as the average expenditure per hospitalization. Statistical analysis (Kruskal–Wallis) confirmed that there were significant differences in the total expenditure across states (p < 0.01). However, the statistical difference among states in terms of the average expenditure per hospitalization ratio became non-significant (p = 0.406) when only crude ratios were compared. This finding highlights the need for multivariate modeling to disentangle the effects of clinical complexity, region, and diagnosis.

**Table 4:** Distribution of hospitalization expenditures for headache disorders across Brazilian states in BRL.

State	Hospitalizations (n)	Expenditures per hospitalization (BRL)^a^	Total expenditures (BRL)^b^
São Paulo	30,824	367.27	11,320,760.02
Ceará	9,974	886.51	8,842,013.26
Paraná	17,937	489.09	8,772,820.05
Pernambuco	8,659	640.87	5,549,334.05
Minas Gerais	10,619	338.36	3,593,078.84
Santa Catarina	7,844	427.21	3,351,008.14
Rio Grande do Sul	7,940	398.22	3,161,847.12
Pará	3,911	702.11	2,745,933.29
Bahia	4,609	344.88	1,589,542.14
Maranhão	6,503	176.65	1,148,780.07
Rio de Janeiro	2,463	365.77	900,893.56
Paraíba	877	675.59	592,490.72
Goiás	2,766	191.28	529,075.57
Espírito Santo	1,584	300.23	475,570.28
Federal District	1,665	263.77	439,174.09
Mato Grosso do Sul	1,099	316.86	348,225.01
Tocantins	1,089	299.62	326,291.52
Mato Grosso	1,188	242.83	288,478.84
Rondônia	1,100	215.10	236,605.61
Alagoas	615	266.58	163,946.06
Piauí	818	174.59	142,815.42
Amazonas	591	234.13	138,373.68
Sergipe	370	312.55	115,642.21
Roraima	595	180.09	107,151.84
Rio Grande do Norte	231	426.69	98,564.51
Acre	433	189.26	81,948.90
Amapá	96	182.97	17,565.50

Note: data obtained from the Hospital Information System of the Brazilian Unified Health System, adjusted for inflation. Brazilian states are classified according to the *Instituto Brasileiro de Geografia e Estatística* (Brazilian Institute of Geography and Statistics). All monetary values are expressed in Brazilian Reais (BRL).

^a^ p = 0.406.

^b^ p < 0.01; *Kruskal-Wallis*.

### Factors Associated with Average Expenditure

The multivariate modeling using the GLM with Gamma family and log link function (Table 5) identified the factors independently associated with the value of hospitalization expenditure. Results are presented as Average Expenditure Multipliers (e^beta^) relative to the reference categories (ICD G43.9, Central-West, 18–59 years, male sex).

**Table 5:** Factors associated with average expenditure per hospitalization (GLM Gamma-Log).

Associate factors	Average expenditure multipliers (eβ)		
	Principal	Sensitivity	Robustness
	229.757^a^	249.179^a^	292.041^a^
Northeast	1.494^a^	1.483^a^	1.519^a^
North	1.105^c^	1.079	1.032
Southeast	1.402^a^	1.408^a^	1.424^a^
South	1.737^a^	1.738^a^	1.728^a^
> 60	1.21^a^	1.218^a^	1.108^a^
< 18	0.92^c^	0.938^d^	0.885^a^
Female	0.939^c^	0.946^d^	0.941^b^
Length of stay	1.17^a^	1.147^a^	1.119^a^
2009	1.062	1.059	1.069
2010	1.086	1.078	1.062
2011	1.032	1.022	1.018
2012	0.993	0.974	0.964
2013	0.922	0.912	0.892^d^
2014	0.853^c^	0.84^c^	0.832^b^
2015	0.864^d^	0.852^c^	0.847^b^
2016	0.805^b^	0.797^b^	0.797^a^
2017	0.863^d^	0.851^c^	0.845^b^
2018	0.889	0.872^d^	0.862^c^
2019	1.052	1.047	0.866^c^
2020	1.001		0.91
2021	1.043		0.88^d^
2022	0.923	0.922	0.804^a^
2023	0.947	0.947	0.889^d^

Note: the model uses average expenditure per hospitalization (total deflated expenditure/number of hospitalizations) as the dependent variable (e^β).

Reference categories: the omitted reference categories are: ICD Diagnosis: G43.9 (Migraine, unspecified); region: Central-West; age group: 18–59 years; sex: male; year: 2008.

^a^ p < 0.001.

^b^ p < 0.01.

^c^ p < 0.05.

^d^ p < 0.1.

The principal diagnosis was the strongest determinant of average expenditure per hospitalization. ICD G44.1 (Vascular headache, not elsewhere classified) recorded the highest multiplier, with average expenditure 247.2% higher (multiplier = 3.47, p < 0.001) than the reference (CID G43.9). G44.0 (Cluster headache) and G43.2 (Status migrainosus) also presented average expenditures of 191.7% and 124.8% higher, respectively.

The average length of stay (days) was a crucial factor, resulting in a 17% increase in the average expenditure for each additional day (multiplier = 1.17; p < 0.001). The 60 years or older group also showed average expenditure 21% higher (multiplier = 1.21, p < 0.001) than the reference group (18–59 years).

Regional variation in average expenditure remained highly significant, even after controlling for diagnosis, age, and days of stay. The South and Southeast regions recorded the highest multipliers: the South region presented an average expenditure 73.7% higher than the Central-West (multiplier = 1.737, p < 0.001). The Southeast region, with a multiplier of 1.402 (p < 0.001), indicated an average expenditure of 40.2% higher (Table 5).

Regarding sex, the expenditure multiplier for females was 0.939 (p < 0.05), indicating that, on average, female hospitalizations cost 6.1% less than male hospitalizations, after adjustment for all factors.

### Robustness and Sensitivity Analysis

The robustness (exclusion of expenditure outliers) and sensitivity (exclusion of the 2020 and 2021 Covid-19 pandemic years) analyses confirmed the validity and consistency of the findings. The hierarchy and significance of the CID and Region multipliers were maintained across all models (Table 5).

## DISCUSSION

This study highlights the substantial burden imposed by headache-related hospitalizations on the SUS, particularly among women and individuals of working age. These results are consistent with previous studies that emphasize the higher prevalence of migraine among women, particularly during reproductive years, when they often experience more frequent and disabling attacks^16,17^. A study conducted in Pelotas, Rio Grande do Sul, with a sample of patients aged 20–64 years, found a migraine prevalence rate of 10.7%, with higher rates among women^18^.

While migraine is most prevalent between the ages of 25 and 55^19^, aligning with our finding of the highest absolute expenditure volume in the 19–59 age group, our multivariate results (GLM, Table 5) showed a critical distinction in the unit burden. Specifically, the 60 years or older group registered 21% higher expenditure per hospitalization compared to the adult reference group. This pattern aligns with previous findings showing that hospitalization costs tend to increase with advancing age, partly due to greater clinical complexity, longer length of stay, and higher prevalence of comorbidities among older adults^20^.

The descriptive analysis of temporal trends (Figure 1) showed peaks in expenditure in the Northeast (2021) and South (2019) regions. Although the formal linear trend analysis was not statistically significant, the North and Northeast regions exhibited increases in hospitalization expenditure over time. This rise, coupled with the finding of the longest median hospital stays in the Northeast and North regions, suggests a possible inequity in access to timely or specialized care. These regions face persistent inequalities in health service utilization^21^. While some studies indicate that higher-income groups may consume more health services overall, the pattern observed here—long stays in the North/Northeast—points to persistent difficulty in accessing effective early management for severe headache disorders^21^.

This is often reflected in health economics, where higher-income groups consume more preventive or outpatient services, while the reliance on and use of hospital services tends to increase as income decreases^22^. For headache, this pattern may indicate that hospitalization becomes the necessary recourse only when the disease has reached a more refractory stage due to deficiencies in specialized outpatient care.

Moreover, migraine and more frequent attacks have been associated with lower socioeconomic status^23^. A 2019 study analyzed regional and social health inequalities in Brazil between 1998 and 2013, revealing that the North and Northeast regions had the lowest percentages of individuals aged 18–59 who rated their health status as “good” or “very good” compared to other regions of the country^24^. Additionally, a study by Viacava et al.^25^ showed that headache has a greater impact on the daily lives of individuals from the North and Northeast regions, those of mixed race (pardo), with low educational attainment and lower income.

The predominance of females in all ICD codes, except for G44.3 (post-traumatic chronic headache), also supports the literature that indicates a higher prevalence in women across most classifications^26^. The identification of status migrainosus (G43.2), vascular headache (G44.1), and cluster headache (G44.0) as the most expenditure-intensive diagnoses and with the longest hospital stays highlights a critical point for public health. The financial weight of these conditions is likely a reflection of their clinical refractoriness. For G43.2 (Status migrainosus), the prolonged duration is directly linked to the need for intensive, sequential intravenous therapies to break the pain cycle. Cluster headache (G44.0) requires highly specialized management due to its intensity and refractoriness to conventional treatment^27^. Similarly, Vascular headache (G44.1) often necessitates extensive neuroimaging and resource use to rule out severe differential diagnoses, such as arterial dissection or stroke^28^.

Regional analysis revealed a dissociation between absolute volume and structural cost burden. While São Paulo concentrated on the highest crude expenditure and hospitalization volume, Ceará recorded the highest average cost per event. On the other hand, the multivariate model described that the South and Southeast regions had the highest unit cost multipliers, suggesting a higher intensity of resource use or better infrastructure in these more developed areas. This finding contrasts with the fact that many of the states with the highest crude unit expenditures (Ceará, Pará) are located in the Northeast and North. These regions are consistently characterized by lower health status assessments, lower educational levels, and poor socioeconomic conditions, which are associated with a higher burden of disease^29^. The juxtaposition of low crude unit expenses (São Paulo) and high adjusted structural expenses (South/Southeast) validates our approach, emphasizing that absolute volume (São Paulo) should not be conflated with the structural expenses burden (South/Southeast) when assessing resource allocation.

Based on publicly available data, this study is subject to inherent limitations. First, the expenditure analysis reflects only the SUS payer perspective, excluding private sector costs and indirect economic losses, thereby underestimating the full economic burden of the disease. Second, potential issues of underreporting and registration errors inherent to administrative databases exist. However, the large volume of data collected provides robust estimates representative of SUS utilization nationwide. Most notably, the findings are representative of the SUS reality, not the total Brazilian healthcare system. Importantly, to the best of our knowledge, no previous national study has examined headache-related hospitalizations in Brazil using such a comprehensive and methodologically robust approach.

This study highlights the high total economic expenditure burden of headaches on the SUS, as well as the greater volume of burden on women and adults of productive age. Most importantly, the GLM results confirmed the persistence of significant structural regional variation in average expenditure. The high average expenditure multipliers in the South/Southeast and the long median stays in the North/Northeast point to structural inequalities in the provision and timeliness of care. These findings reinforce the urgent need for public policies that optimize headache management, reduce high-cost hospitalizations, and address regional disparities in care provision.

## Data Availability

The data supporting the findings of this study are publicly accessible through DATASUS: http://www.datasus.gov.br

## Footnotes

a Available from: https://github.com/rfsaldanha/microdatasus
